# Optimization of Extraction Parameters for Phenolics Recovery from Avocado Peels Using Ultrasound and Microwave Technologies

**DOI:** 10.3390/foods14142431

**Published:** 2025-07-10

**Authors:** Lorena Martínez-Zamora, María Carmen Bueso, Mathieu Kessler, Rosa Zapata, Perla A. Gómez, Francisco Artés-Hernández

**Affiliations:** 1 Postharvest and Refrigeration Group, Department of Agricultural Engineering & Institute of Plant Biotechnology, Universidad Politécnica de Cartagena, 30203 Cartagena, Murcia, Spain; lorena.martinez@upct.es (L.M.-Z.); rosa.zapata@upct.es (R.Z.); perla.gomez@upct.es (P.A.G.); 2 Department of Food Technology, Nutrition, and Food Science, Faculty of Veterinary Sciences, University of Murcia, 30071 Espinardo, Murcia, Spain; 3 Department of Applied Mathematics and Statistics, Universidad Politécnica de Cartagena, 30202 Cartagena, Murcia, Spain; mcarmen.bueso@upct.es (M.C.B.); mathieu.kessler@upct.es (M.K.)

**Keywords:** *Persea americana*, polyphenols, green extraction technologies, revalorization, procyanidins, response surface methodology

## Abstract

Background: Avocado (*Persea americana*) peels account for ~20% of the fruit weight and are rich in bioactive compounds, offering significant revalorization potential. This study optimized the extraction parameters of phenolics using ultrasound- (UAE) and microwave-assisted technologies (MAE) with a Central Composite Design (CCD). Methods: The extraction variables included EtOH concentration (0–100%), temperature (13–47 °C for UAE and 55–95 °C for MAE), and time (3–37 min for UAE and 3–27 min for MAE). Total antioxidant capacity (TAC) and total phenolic compounds (TPC) were measured, while individual phenolics were analyzed via HPLC/MS. Results: EtOH concentration was the most influential variable, with optimal conditions involving 94.55% EtOH and moderate temperatures over short times (45 °C for 5 min in UAE and 67 °C for 12 min in MAE). Both techniques yielded comparable results for effective conditions, though MAE required higher temperatures and longer times. In this sense, the data show that UAE extracted higher concentrations of procyanidins (+15%), demonstrating superior performance using a lower time and temperature, making it more efficient. Conclusions: UAE and MAE effectively extract antioxidants, promoting sustainability in the agri-food sector.

## 1. Introduction

Avocado (*Persea americana*) is a fruit-bearing species within the Lauraceae family, classified under the Plantae kingdom, Laurales order, and Persea genus. Native to Mexico and Central America, it thrives in tropical and subtropical climates. Among members of the Lauraceae family, avocado stands out as the most commercially significant and widely consumed fruit. The Persea genus comprises over 150 species, with approximately 70 found in the warmer regions of the Americas. The fruit exhibits diverse morphological characteristics, varying in shape (pyriform, ovate, or globular), surface texture (smooth or rough), and skin thickness (thin to thick) [1,2].

One key industrial use of avocados is in the production of guacamole or avocado oil, during which the peel and pit are separated from the pulp and collected as by-products. An important problem to solve in the horticultural industry is the huge amount of by-product generated. Around 140 M tonnes of losses were produced in 2010, while in recent years, 600 M tonnes have been generated worldwide [3,4]. In the case of avocados, the percentage of peels (16–21%) and seeds (18.6%) represent most of its by-products [5,6,7].

Avocado by-products contain phenolic compounds such as quercetin glycosides, A- and B-type procyanidin dimers, A-type procyanidins (dimer and trimers), catechin, caffeoylquinic acid, and coumaroyl-quinic acid [8]. The peel of the Hass avocado makes up 11–17% of the total fruit’s weight and contains several bioactive compounds, such as phenolic compounds (phenolic acids, flavonoids, and phenolic alcohol derivatives) and organic acids [1,5].

Avocado extracts have been used in traditional medicine to treat several diseases since they possess many interesting healthy and preservative properties: larvicidal, antifungal, antimicrobial, antioxidant, antiprotozoal, antidiabetic, antihypertensive, hypocholesterolemic, and antimycobacterial activity as well as lipid and protein oxidative inhibition [1,5]. For recovering the highest amount of desired bioactive compounds from the plant material, different extraction methods have been studied. In this sense, methods for the extraction of phenolics from non-edible parts of fruits and vegetables, such as peels, that are continuously exposed to sunlight, which promotes the synthesis of phenolics, have been developed in last years to create new ingredients rich in higher concentrations of healthy promoting compounds, which justify the selection of these by-products in the present work [9,10].

The use of bioactive compounds as ingredients is subjected to the methods employed for their extraction from plant materials, followed by their chemical characterization, separation, isolation, purification, and potential application in functional foods and nutraceuticals. These techniques can be broadly categorized into conventional and non-conventional methods. Conventional methods typically involve solid-phase or liquid–liquid extraction techniques, such as Soxhlet extraction or maceration [11]. These processes commonly use organic solvents like chloroform, ether, petroleum ether, hexane, or acetone [5,12,13]. By contrast, ‘Green’ or non-conventional techniques include supercritical fluid extraction (SFE), subcritical CO_2_ extraction (SCE), pressurized liquid extraction (PLE), ultrasound-assisted extraction (UAE), enzyme-assisted extraction (EAE) and microwave-assisted extraction (MAE), among others. Such methods use environmentally friendly solvents that can be substituted for traditional organic solvents such as H_2_O or EtOH [5,12,13,14]. The advantages of these technologies are that they are environmentally friendly, economical, and the processes can be quickly completed with high reproducibility, low solvent consumption, minimized environmental impact, high extraction purity, and low energy consumption.

In recent years, modeling has become an effective tool to reduce the costs and time associated with conducting extensive experiments. Mathematical modeling makes it possible to simulate, optimize, design, and control processes through the application of predictive tools such as Response Surface Methodology (RSM) to analyze the interactions between studied variables. The Central Composite Design (CCD) method contains an integral factorial or fractional factorial design with central points that are augmented with a group of star/axial points that allow the estimation of curvature [15,16], and it was selected as the model used in this work.

This study aimed to optimize the extraction of phenolic compounds from avocado peel by-products using UAE and MAE. The effects of key process variables—temperature, extraction time, and solvent composition (EtOH and H_2_O)—were systematically evaluated. Optimization was carried out using the RSM. A distinctive aspect of this work lies in the comprehensive presentation of the experimental design, including the justification of the selected model, definition of independent variables and their tested ranges, assessment of model validity, and detailed explanation of the optimization strategy. Furthermore, this emerging research area has only been investigated in avocado by-products through the spectrophotometric analysis of their total bioactive compounds, whereas the present study targets the extraction and identification of individual phenolic compounds with potential health benefits, such as epicatechin and procyanidins. Furthermore, as the main novelty compared to previous studies, this work simultaneously compares both extraction technologies while evaluating the influence of key extraction parameters such as solvent, time, and temperature.

## 2. Materials and Methods

### 2.1. Vegetal Material

The plant material was ‘Hass’ avocados, provided by the company P.I. Trops (Málaga, Spain), from which the pulp was used to obtain guacamole, the seeds were used in another experiment, and the peels were used as by-products in this work. Freeze-drying (72 h, −20 °C, and 400 mbar in a Telstar^®^ LyoBeta, Terrassa, Spain) and grinding pre-treatment steps were applied to ensure the raw material used for extraction was both stable and homogeneous. The obtained powder was kept in vacuum bags in dark conditions at 20 °C.

### 2.2. Experimental Design

The RSM comprises various experimental design strategies, among which CCD was selected due to its widespread application in optimizing the extraction of bioactive compounds from horticultural residues. Two separate CCDs were constructed to fit each extraction technique: UAE and MAE. Based on prior studies [17,18], several process parameters were held constant in both designs: (i) particle size (<56 µm), (ii) solid-to-liquid ratio (1:30, *w*:*v*), (iii) UAE amplitude (65%), (iv) duty cycle (30:30), (v) US frequency (20 kHz). Table 1 outlines the independent variables used for UAE [T: temperature (13–47 °C); t: time (3–37 min); and S: solvent (0–100% EtOH in H_2_O), with a nominal power of 700 W] and MAE [T: temperature (55–95 °C); t: time (3–27 min); and S: solvent (0–100% EtOH in H_2_O), with a nominal power of 1900 W], including their respective levels (−α, −1, 0, +1, +α).

The chosen levels were selected from the prior literature [17,18] and insights from preliminary, unpublished experiments. The −1 and +1 levels were established according to the operational limits of the ultrasound equipment and guided by expectations based on existing research. In this design, the response variables corresponded to the phenolic compounds identified. The CCD was structured into three blocks with randomized treatment combinations, and consistent with previous studies, four replicates of the central point were included. Table 1 summarizes the experimental design. An additional block was incorporated including extra central point replicates to validate the findings. Twenty experimental runs were performed in a randomized order, as shown in Table 2.

### 2.3. Phenolic Compound Ultrasound- and Microwave-Assisted Extraction and Quantitative Analysis

UAE was performed using a probe-type sonicator (Fisherbrand™ Q705, Madrid, Spain; dimensions: 387 × 203 × 216 mm) equipped with a 12.5 mm diameter tip. The device operated at 110 V, with a maximum power output of 700 W and a frequency of 20 kHz. For each extraction, 1 g of freeze-dried avocado by-product was mixed with 30 mL of solvent in Falcon tubes. These tubes were then placed in a 500 mL glass container filled with water, and the desired temperature was maintained using a combination of ice and a hot plate, following the conditions outlined in Table 2. The ultrasonic probe was inserted directly into the Falcon tube, while the temperature sensor was placed in the surrounding water bath.

The MAE was carried out using a microwave device (Milestone, ETHOSTM UP, 68 Italy) equipped with an SK-10 rotor with larger upper-edge TFM vessels (P/N 34040), including 9 standard segments and 1 reference segment for temperature and pressure control. Samples of 1 g of freeze-dried avocado by-products and 30 mL of solvent were added into the TFM vessels. Once every vessel was properly fitted in the rotor, the extraction started following the conditions described in Table 2.

After extraction, the samples were centrifuged at 4000 rpm for 10 min to separate the solids from the liquid phase. The resulting supernatant was then filtered using 0.2 µm PTFE syringe filters (Millipore, Bedford, MA, USA) prior to chromatographic analysis. The filtered extracts were stored at −80 °C until further use. Phenolic compounds were analyzed and quantified using an Agilent 1200 HPLC system (Santa Clara, CA, USA) equipped with a G1311B quaternary pump, G1329B autosampler, and G1316A column compartment and coupled to a 6420 triple quadrupole mass spectrometer (QqQ) with an electrospray ionization (ESI) interface.

The chromatographic separation was carried out on a Luna Omega C18 (2.1 × 100 mm, 3 µm particle size) reverse-phase column (Phenomenex, Torrance, CA, USA). The mobile phase was composed of solvent A (0.1% CH_2_O_2_ in H_2_O) and solvent B (C_2_H_3_N). The system operated in negative ionization mode, with a sample injection volume of 5 µL and column temperature set to 40 °C. The gradient elution began with 10% B for 5 min, increased to 25% B over the next 5 min, followed by a linear ramp to 50% B in 7 min, and then to 95% B in 14 min. After holding at 95% B for 7 min, the gradient returned to 25% B in 23 min, with a total run time of 63 min. The flow rate was maintained at 0.2 mL min^−1^.

**Figure 1:**
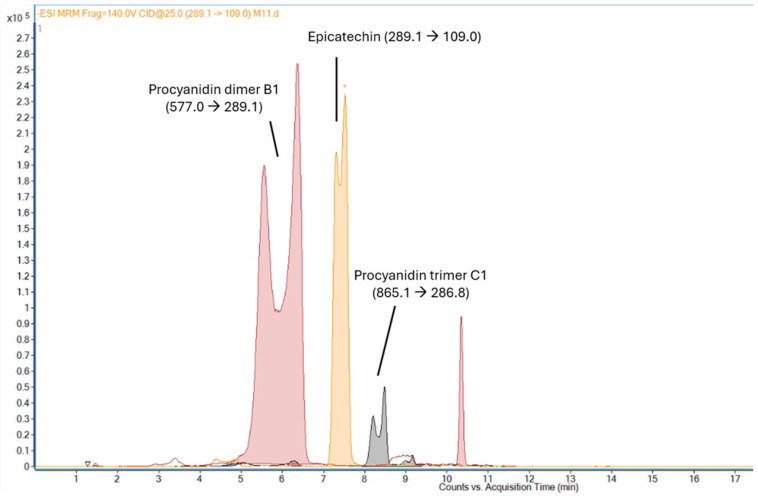
HPLC-MS signals obtained after Multiple Reaction Monitoring (MRM) of identified compounds in UAE and MAE of avocado peel by-products.

Mass spectrometry parameters included a gas temperature of 350 °C, a capillary voltage of −4000 V, a nebulizer pressure of 40 psi, and a gas flow of 11 L min^−1^ (Figure S1). Phenolic compounds were identified according to the confidence level criteria described by Schymanski et al. [19], based on retention time and MS/MS fragmentation patterns, and confirmed using analytical standards or published reference data (Figure 1). Multiple Reaction Monitoring (MRM) was used to quantify the bioactive compounds, selecting a specific transition for each molecule (Table S1), and results were shown as mg kg^−1^ dried weight (dw) of sample using commercial standards as precursors of such compounds (relative information to quantification is shown in Table S2).

### 2.4. Total Phenolic Content and Total Antioxidant Capacity

Total phenolic content (TPC) analysis was carried out according to Singleton and Rossi’s method [20] with some modifications. Briefly, 19 µL of the extract was mixed with 29 μL of 1 N Folin–Ciocalteu reagent. After 3 min in darkness, 192 μL of 0.4% Na_2_CO_3_ and 2% NaOH were added, and the mix was incubated for 1 h in darkness at room temperature. The absorbance of each sample was recorded at λ = 750 nm in an Infinite PRO 2000 microplate reader (Tecan Trading AG, Männedorf, Switzerland). The TPC was expressed as mg of gallic acid equivalent (GAE) kg^−1^ dw (y = 0.197x − 0.008; R^2^ = 0.997).

The total antioxidant capacity (TAC) was analyzed following the 2,2′-azino-bis-(3-ethylbenzothiazoline-6-sulfonic acid) radical scavenging method (ABTS) [21]. For that assay, 200 μL of the activated 32 μM ABTS solution was added to 11 μL of the extract and incubated for 30 min at room temperature in darkness, when the absorbance was measured at λ = 414 nm. Trolox standard (y = 15.70x + 0.017; R^2^ = 0.993) was used to calculate TAC, which was shown as mg of Trolox equivalent (TE), kg^−1^ dw.

### 2.5. Statistical Analyses

Statistical analyses, data processing, and visualization were carried out using R software (R Core Team, 2023). To reduce the influence of uncontrolled sources of variability, the experimental runs were conducted in a randomized sequence. The predictive accuracy of the model for optimized phenolic extraction was performed by comparing the estimated values under optimized conditions with those obtained experimentally. The goodness of fit for the response surface model was evaluated through the R-squared metric (Tables S3 and S4). Specific residual plots were also examined, and a Shapiro–Wilk normality test was conducted, showing no significant violations of the statistical assumptions of the model.

## 3. Results and Discussion

UAE and MAE have evolved as efficient extraction methods aimed at reducing resource consumption, such as time, solvent use, and energy, making them more sustainable for industrial applications. In this work, EtOH:H_2_O mixtures were employed as extraction solvents, chosen for their recognized status as generally safe (GRAS).

### 3.1. Central Composite Design Center Value

Extraction efficiency is affected by parameters such as temperature, time, solvent composition, and solvent–sample ratio, among others, which can influence efficiency independently or interact with each other [9].

The centers of the present design were based on the scientific literature, specifically the optimized conditions for phenolic extraction. Therefore, the selected centers were 30 °C, 20 min, 50% EtOH (30 s pulse duration), and 65% amplitude for UAE [17,22,23,24,25], and 75 °C, 15 min, and 50% EtOH for MAE [17,18]. The experimentally verified response variables for UAE and MAE under such conditions were procyanidin (C1 and B1), and epicatechin, whose obtained values were 4.92 and 5.42 g kg^−1^, 31.13 and 32.96 g kg^−1^, and 7.43 and 7.99 g kg^−1^, respectively (Table 2). However, our results can be only compared to those obtained by Martínez-Gutiérrez [22], who obtained ~0.23 g epicatechin kg^−1^ extract by EtOH and water sonication by bath, as the rest of the authors in the literature optimized their extractions based on the spectrophotometric analysis of TPC and TAC. This demonstrates that continuous US application is more effective for extracting bioactive compounds when applied directly to the solid–solvent mixture using a probe, rather than through an ultrasonic bath [26,27]. Furthermore, the pulsed ratio used in the present experiment (30 s on 30 s off) was useful to control the temperature and to reduce the number of cavitation bubbles while increasing the intensity of their implosion to enhance the extractive capacity of this technology [26]. Furthermore, using a sonotrode, Razola-Díaz et al. [25] reported a yield of approximately ~20 g kg^−1^ dw of total procyanidins under their optimized conditions (65% EtOH, 25 min, and 65% US amplitude), which was slightly lower than our results. This difference may be attributed to the much higher solid-to-solvent ratio used in their study (1:200; *w*:*v*) compared to ours (1:30; *w*:*v*), as they used the same cultivar from the same Spanish region (Málaga). Also, variations in the obtained results can be due to the cultivar used, the by-product type, the pre-treatment applied to the studied samples (drying method, particle size), or the solid–liquid ratio. Martínez-Gutiérrez [22] used the same avocado variety, but their pre-treatments (no drying), ratio (1:10) (*w*:*v*), and extraction procedure were different (15 min, frequency 40 KHz, power 150 W, Ultrasonic Cleaner D150). In fact, a larger liquid-to-solid ratio allows for the greater transfer of compounds into the liquid solvent. This is due to a favorable concentration gradient during diffusion and the reduced viscosity of the system, which enhances the cavitation effect, allowing a higher extraction yield [26].

### 3.2. Extraction, Identification, and Quantification of Phenolic Compounds

**Figure 2:**
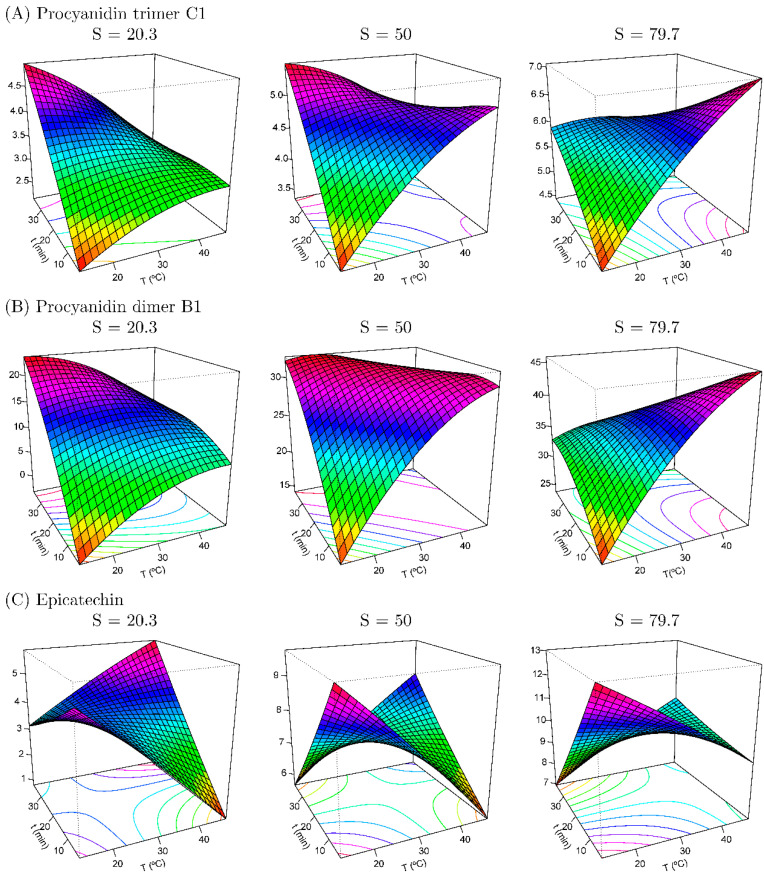
Response surface maps of procyanidin C1 (**A**), procyanidin B1 (**B**), and epicatechin (**C**) (g kg^−1^) from UAE of avocado peels according to concentration of EtOH used as solvent (S).

**Figure 3:**
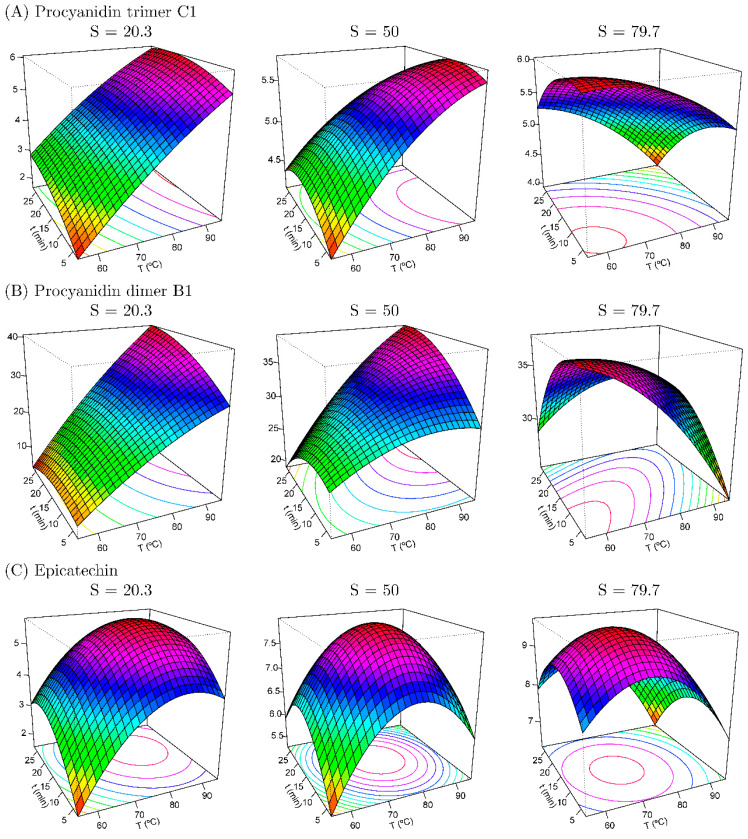
Response surface maps of procyanidin C1 (**A**), procyanidin B1 (**B**), and epicatechin (**C**) (g kg^−1^) from MAE of avocado peels according to concentration of EtOH used as solvent (S).

**Figure 4:**
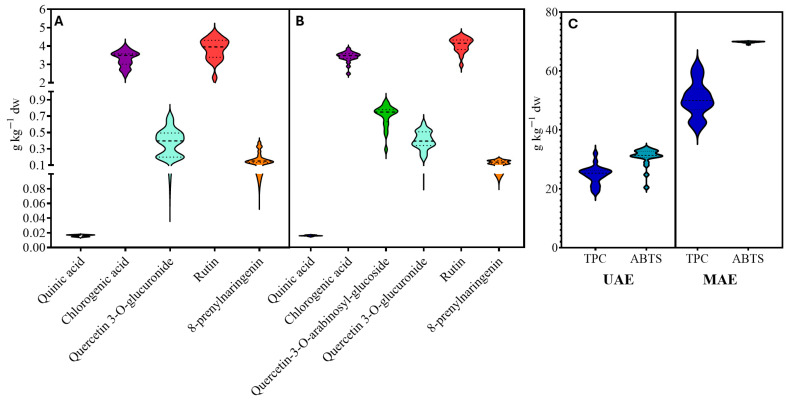
Violin plot with identified phenolics from UAE (**A**) and MAE (**B**), and total phenolic compounds (TPC) and total antioxidant capacity (TAC) (by ABTS) (**C**) results of avocado peels.

Table S3 represents the least squares estimation of the regression coefficients based on the data obtained, determining that the solvent variable (X3) influences the extraction of the majority bioactive compounds with 95% confidence. The RSM has proven to be a powerful tool for the optimization of extraction procedures thanks to the possibility of evaluating the interaction effects between variables on the response [9] without the need to analyze many replicates or repetitions. Table 3 shows the optimum conditions and values reported for those variables that adapted to the proposed RSM model with significance and R^2^ > 0.88 (Figure 2 and Figure 3). Figure 4 shows the obtained data for TPC, TAC (by ABTS), and the other quantified compounds of avocado peel extracts obtained by UAE and MAE.

In this sense, better results were reported in both extraction methods when using higher concentrations of EtOH as an extractant, with the optimum conditions for the extraction being 45 °C and 5 min for UAE, while for MAE they were 67 °C and 12 min (Table 3).

A comprehensive analytical characterization of phenolic compounds in avocado peel was performed, allowing the identification of the extracts. The analysis reported the identification of 11 major compounds (Table S1 and Figure S1). The identification was performed on the basis of their retention time, mass, and fragmentation (Table S1), based on previous literature [6,9,18,28]. In the study performed by Figueroa et al. [9], 61 compounds were identified in avocado peel extract with a HPLC-DAD-ESI-QTOF-MS system, representing several families of compounds. The most prominent groups were procyanidins, flavonols, hydroxybenzoic acids, and hydroxycinnamic acids. Among these, condensed tannins were the largest group, with 17 procyanidins, where dimeric A and B procyanidins appeared in different isomeric forms. In the current study, HPLC-ESI-QqQ-MS allowed the identification of key compounds, including B1 dimer and C1 trimer procyanidins; organic acids such as quinic, cholorogenic, and hydroxy-oxo-octadecenoic; and flavonoids as epicatechin, quercetin-3-O-arabinosyl-glucoside, and quercetin 3-O-glucuronide, rutin, and 8-prenylnaringenin. These compounds are known for their strong antioxidant properties due to their abundance of hydroxyl groups, which can neutralize free radicals, as reported in previous research [28,29,30]. In this sense, Tremocoldi et al. [29] also identified high concentrations of procyanidin B2 and epicatechin as major compounds in the peel of Hass variety avocados. More recently, Razola-Díaz et al. [25] reported approximately half the extraction yield of procyanidins (~21 mg g^−1^ dw) compared to our results, using 60% EtOH for 30 min and a US amplitude of 70%. This difference demonstrates that our experimental conditions successfully optimized the extraction of procyanidins from the same food matrix using identical samples (Hass cv.) from Málaga. Although this improvement may be largely attributed to the optimized parameters in our study, some variability could also be explained by the ratio used (1:30 w:v in our case, and 1:200 *w*:*v* in the case of Razola-Díaz et al.) [25] and seasonal differences, as their experiments were conducted in 2020, while ours took place in 2024.

Figure 1 shows the obtained chromatogram, where the first peak corresponds to procyanidin B1, the second peak to epicatechin, and the third one to procyanidin C1. The quantification of the areas was carried out based on the possible presence of isomers of the same molecular weight that generate a double peak, but which are equally interesting for studying and quantifying their antioxidant properties. According to literature sources, the presence of procyanidin dimers and trimers, which belong to the class of proanthocyanidins, could be confirmed [23,30,31]. The procyanidin C1 trimer, procyanidin B1 dimer, and epicatechin were identified considering their *m*/*z* [M-H]- and retention time through comparison with data present in the literature [22,28,32] and comparing their retention time with a standard made of the primary standard molecules (procyanidin B1 dimer and epicatechin). Therefore, the concentration of significant compounds (g kg^−1^ dw avocado sample) is shown in Table 2 (for UAE and MAE). The highest concentrations of epicatechin and procyanidins were obtained in samples extracted under conditions with a high percentage of EtOH, temperatures between 30 and 45 °C, and extraction times of 10–30 min for UAE, and temperatures of 60–75 °C and extraction times of 15–22 min for MAE. Therefore, the use of EtOH (>80% EtOH) allowed the extraction of high concentrations of bioactive phenolic compounds.

Both in the quantification of the identified flavonoids, as well as TAC and TPC, higher values are observed in conditions where the % EtOH variable is high, while the temperature and time variables fluctuate, thus justifying the conclusion that EtOH is the variable that directly affects the extraction yield of the bioactive compounds. However, according to the literature, increasing the extraction time increases the extractive yield. Similarly, increasing the temperature facilitates the extraction process of the analytes. However, a long extraction time combined with high temperatures may stimulate the hydrolysis of total phenolic compounds, as suggested in Posta et al. [23].

Figure 2 and Figure 3 show the response surface maps for those variables and interactions that were significant (*p* ≤ 0.05) in the identification of procyanidins and epicatechin for the UAE and MAE of avocado peels, respectively. Table 3 shows the optimized values according to the CCD for the extraction of the major bioactive compounds from avocado peel using UAE, being for the procyanidins (both trimer C1, 7.793 g kg^−1^ and dimer B1, 49.04 g kg^−1^) a percentage of 94.55% of EtOH at 45 °C for 5 min, and for epicatechin 94.55% EtOH, 15 °C, and 5 min (13.63 g kg^−1^). Furthermore, for epicatechin extracted by MAE, the optimum value was 10.29 mg kg^−1^ obtained at 94.55% EtOH, 67.32 °C, and 12.2 min.

Figure 2 presents response surface plots for the UAE of procyanidin dimer, trimer, and epicatechin, with R^2^ values of the modeling of 0.888, 0.928, and 0.929, respectively, indicating the good fit of the models (Table 3). A detailed description of the obtained results is described below:

- At the −1 level of the solvent (20.3% EtOH), the temperature and extracted procyanidins increased proportionally while the epicatechin content decreased, and a similar behavior was shown with the time, as the procyanidin extraction increased with longer times while the epicatechin was kept constant. At elevated temperatures (45 °C), differences in extraction times resulted in minimal variation (less than 10%). In contrast, at lower temperatures, an increase in extraction time led to a notable rise in the levels of extracted procyanidins B1 and C1. In fact, the maximum concentration of these compounds was observed under these low-temperature, extended-time conditions. At lower temperatures, the time did not affect the extraction of epicatechin.
- At the 0 level of the solvent (50% EtOH), with shorter extraction times, a decrease in extracted procyanidins was observed as the temperature decreased. By contrast, epicatechin extraction increased at lower temperatures and shorter times (violet colors). Variations throughout incubation time were not relevant (≈10%) at high temperatures (45 °C) for procyanidin extraction, while epicatechin showed the lowest extraction ratio, being highly affected by the time and increasing proportionally to this parameter.
- At the +1 level of the solvent (79.7% EtOH), the tendency followed with shorter times was like those of the results obtained at the −1 and 0 levels for procyanidins, whereas the temperature and time decreased, the extracted procyanidin content also decreased proportionally. By contrast, epicatechin extraction followed the contrary behavior, having higher yields at lower temperatures and shorter times. In fact, the same occurred at high temperatures (45 °C) and short times, obtaining the highest extraction yields for procyanidins and lower yields for epicatechin. Therefore, scarce variations were found between times (<10%) for procyanidin content, while the same lack of variation was found with temperature changes in epicatechin extraction. In this sense, with nearly these conditions of EtOH percentage, Table 3 shows a maximum content of procyanidin (7.793 and 49.04 g kg^−1^ dw for trimer C1 and dimer B1) at this % EtOH of 94.55% (45 °C and 5 min), being ~58% higher than our center. Similarly, the maximum epicatechin content (13.63 g kg^−1^ dw) was extracted with 94.55% EtOH, at 67.32 °C, and after 12.2 min, which supposes an increase of ~83% compared to the established centers.

On the other hand, the R^2^ of the modeling for the MAE (Figure 3) of epicatechin was 0.920, showing the good fit of the model, while the rest of the variables monitored did not show this goodness of fit (Table 3). As shown, in this case, the centers chosen for the present experiment were 75 °C, 15 min, and 50% EtOH, according to previous findings of Araujo et al. [18] and Trujillo-Mayol et al. [17]. Under these conditions, 7.99 g kg^−1^ of epicatechin was extracted (level 0; S = 50%) (Table 2). This yield increased by approximately 29% when the EtOH concentration was raised to 94.55% and the extraction time (12.2 min) and temperature (67.32 °C) were slightly adjusted (Table 3). These modifications may also contribute to energy savings due to the reduced time and temperature used during MAE. In this sense, at short times (<5 min) and low temperatures (<55 °C) the epicatechin extraction showed a negative tendency, as well as at long times (>25 min) and high temperatures (>95 °C). With this, and corroborating the results obtained by Araujo et al. [18] and Trujillo-Mayol et al. [17], the MAE of phenolic compounds has been optimized, specifically for compounds with high functional properties such as epicatechin, which was not evaluated by the previous authors, which can open a new field of possibilities in the extraction, purification, and use of food by-products in the cosmetics, pharmaceutical, or food industries.

By contrast, in the case of procyanidins, the R^2^ was lower than 0.7, which would mean that the model poorly explained the variability in the data, suggesting a weak correlation between the independent variables (like time, temperature, and solvent ratio) and the yield of procyanidin extraction. For these compounds, there may have been interactions or nonlinear effects not adequately captured by the present model. However, the results indicate that, under these conditions, the extraction of procyanidins is more efficient with EtOHic solvents (94.55% EtOH), short extraction times (4.5 min), and moderate temperatures (57 °C). Notably, since the MW device did not initiate the process below 60 °C, this suggests that US is a more effective technology for extracting these flavan-3-ol compounds, as high temperatures are not required. This is also advantageous for saving energy and reducing costs.

Kinetic parameters related to both degradation and extraction are consistently affected by various operational factors, including temperature, processing time, solvent, ratio, power (for MAE), and amplitude (for UAE). Altering the UAE or MAE parameters can significantly affect the system’s behavior and its extractive yield. To determine if the proposed maximum represents the upper limit of extraction for this method, future research should explore longer extraction times. While increasing the amplitude could be beneficial for UAE, it is crucial to assess energy consumption as an important factor in optimizing conditions. Additionally, it has been found that high amplitudes can lead to the formation of a cavitation bubble layer around the probe tip, which interferes with energy transfer to the extraction medium, thereby lowering the yield [26].

### 3.3. Phenolic Content and Total Antioxidant Activity

As shown in Figure 4, non-significant (*p* < 0.05) differences were detected among observations regarding spectrophotometric analysis (TPC and ABTS) and the concentration of the individual compounds quantified: quinic acid, chlorogenic acid, quercetin-3-O-arabinosyl-glucoside, quercetin 3-O-glucuronide, rutin, and 8-prenylnaringenin. A violin plot was used to visualize the distribution of the data and its probability density (Figure 4). It is similar to a box-plot, with the addition of a rotated kernel density plot on each side, usually smoothed by a kernel density estimator. Typically, a violin plot will include all the data found in a box plot: a marker for the median of the data (thick intermittent line) and a box or marker extending from it indicating the interquartile range (thin intermittent line) and representing the 95% confidence intervals.

Box-and-whisker plots are limited in their visualization of the data, as their visual simplicity tends to obscure significant details about how values are distributed in the data. For example, with box-and-whisker plots you cannot see whether the distribution is bimodal or multimodal, while violin plots include more information and can be much more cluttered than the first.

The violin plot includes each analysis without significant differences, as well as the horizontal width of the box. Commonly, the mentioned spectrophotometric methods (Folin–Ciocalteu and ABTS) are widely used to determine the TPC and TAC. These assays are limited by their non-specific nature, as both are spectrophotometric methods that do not distinguish individual compounds. Although no significant trends were observed in the TPC and TAC results, the assays were influenced by matrix components and may have overestimated antioxidant activity due to the presence of non-phenolic reducing agents. In contrast, the identification of specific phenolic compounds, such as epicatechin and procyanidins, enables a more accurate correlation between molecular structure and potential bioactivity. Therefore, the use of targeted chromatographic techniques provides a deeper understanding of the functional properties of avocado by-products. Notably, significant trends were observed for major compounds extracted from avocado peels, with a strong model fit (R^2^ > 0.88), offering more precise insights into individual phenolic extraction behavior compared to global spectrophotometric methods.

In this study, variations in extraction time, temperature, and EtOH proportion did not lead to notable differences in the recovery of these compounds from avocado peel when using UAE or MAE.

Therefore, due to the richness of the obtained extracts in phenolic compounds such as epicatechin and procyanidin following the optimization process of both extractive technologies, UAE and MAE, these avocado peel extracts could be potentially used in applications as antioxidants and antibacterials in the food and cosmetic industries with promising results [8], although UAE with a probe (or sonotrode) is more recommended due to the use of lower temperatures (<60 °C), as these temperatures cannot be reached or maintained during MAE.

## 4. Conclusions

It was possible to optimize the extraction conditions for the major bioactive compounds identified in avocado peels thanks to the CCD model used in this study. UAE reported the best conditions with 94.55% EtOH, 45 °C, and 5 min to extract procyanidins, and at 15 °C for epicatechin. MAE reported the best efficiency using 94.55% EtOH, 67.32 °C, and 12.2 min for epicatechin. However, since this technology is not effective at controlling temperatures below 60 °C, it is not recommended for the extraction of procyanidins, which did not fit the proposed model. Moreover, using least squares estimation with a 95% confidence level, EtOH concentration was identified as the variable that significantly affected the extraction of bioactive compounds. No changes in the concentration of other minor bioactive compounds and TAC were observed at different extraction temperatures and times. Nevertheless, the extraction of flavonoids from avocado peels by UAE and MAE was optimized, showing that the factor that most influences the extraction is the solvent used, while the rest of the variables do so to a lesser extent. These types of green technologies are useful to reduce food losses within a circular economic scenario and take advantage of the inedible parts of fruits which are rich in health-promoting compounds. Future perspectives should focus on the use of pilot plant equipment for such extraction and subsequent encapsulation before fortifying new foods with such extracted biocompounds. Furthermore, the assessing energy use and cost implications associated with different input conditions would also be essential for informing future decisions regarding industrial-scale implementation.

## Figures and Tables

**Table 1:** Independent variables (temperature, time, and solvent composition) and their corresponding coded levels (−α, −1, 0, +1, +α) used in CCD for UAE- and MAE-based extraction of bioactive compounds from avocado peel.

	Input			Input Levels				
				−α	−1	0	+1	+α
**UAE**	X1	Temperature (°C)	T	13	20	30	40	47
	X2	Time (min)	t	3	10	20	30	37
	X3	% EtOH	S	0	20.3	50	79.7	100
**MAE**	X1	Temperature (°C)	T	55	63	75	87	95
	X2	Time (min)	t	3	8	15	22	27
	X3	% EtOH	S	0	20.3	50	79.7	100

**Table 2:** CCD for input variables and responses for UAE and MAE of main bioactive compounds in avocado peels (g kg^−1^ dw).

		UAE						MAE						
Run Order		Inputs			Responses			Run Order	Inputs			Responses		
		T	t	S	Procyanidin C1	Procyanidin B1	Epicatechin		T	t	S	Procyanidin C1	Procyanidin B1	Epicatechin
Block 1	2	20	30	20.3	4.33	20.66	3.64	4	63	22	79.7	6.02	35.39	9.18
	1	40	10	20.3	3.18	7.60	1.53	3	87	8	79.7	6.78	39.54	9.74
	6	30	20	50.0	5.56	33.81	6.75	5	75	15	50.0	5.79	34.18	8.27
	3	40	10	79.7	6.96	43.64	9.76	2	63	22	20.3	3.08	10.44	3.63
	4	20	30	79.7	6.30	39.97	9.10	1	87	8	20.3	5.42	31.14	5.13
	5	30	20	50.0	4.82	31.86	7.42	6	75	15	50.0	5.45	31.26	7.82
Block 2	2	40	30	20.3	3.39	15.34	3.29	5	75	15	50.0	5.72	32.53	7.93
	1	20	10	20.3	2.94	5.00	3.53	2	87	22	20.3	5.84	35.13	5.94
	6	30	20	50.0	5.38	34.54	8.18	1	63	8	20.3	3.09	10.09	3.45
	4	40	30	79.7	6.51	40.60	9.95	3	63	8	79.7	6.50	40.34	9.10
	3	20	10	79.7	5.87	34.39	12.17	6	75	15	50.0	5.29	31.46	7.41
	5	30	20	50.0	4.49	25.65	6.96	4	87	22	79.7	5.45	42.02	8.65
Block 3	2	47	20	50.0	4.53	30.71	8.15	2	95	15	50.0	4.65	27.07	5.89
	3	30	3	50.0	4.20	28.02	8.16	5	75	15	0.0	3.84	21.50	3.82
	7	30	20	50.0	4.70	31.31	8.14	8	75	15	50.0	5.10	35.96	8.35
	8	30	20	50.0	4.60	29.65	7.12	4	75	27	50.0	4.92	29.34	7.25
	5	30	20	0.0	2.91	3.26	2.08	3	75	3	50.0	4.29	25.73	6.63
	4	30	37	50.0	4.78	26.99	7.48	6	75	15	100.0	4.10	24.63	9.37
	6	30	20	100.0	5.99	34.01	9.90	7	75	15	50.0	5.22	32.32	8.16
	1	13	20	50.0	4.23	23.40	7.07	1	55	15	50.0	4.55	28.53	7.34

T: temperature (°C); t: time (min); S: EtOH (%).

**Table 3:** Model performance indicators (R^2^, *p*-values) and optimal extraction conditions for UAE and MAE of bioactive compounds from avocado peels.

	Compound	R^2^	*p*-Value	Optimized S (% EtOH)	Optimized T (°C)	Optimized t (min)	Optimized Concentration (g kg^−1^)
UAE	Procyanidin trimer C1	0.888	0.00105	94.55	45	5	7.793
	Procyanidin dimer B1	0.928	0.000132	94.55	45	5	49.04
	Epicatechin	0.929	0.000121	94.55	15	5	13.63
MAE	Procyanidin trimer C1	0.624	ns (0.176)	94.55	57	4.5	6.61
	Procyanidin dimer B1	0.67	ns (0.11)	94.55	57	4.5	41.59
	Epicatechin	0.920	0.000215	94.55	67.32	12.2	10.29

ns: not significant.

## Data Availability

The original contributions presented in this study are included in Table 2 and the Supplementary Materials. Further inquiries can be directed to the corresponding author.

## Supplementary Materials

The following supporting information can be downloaded at: https://www.mdpi.com/article/10.3390/foods14142431/s1, Table S1. Identification of phenolics and other polar compounds in UAE and MAE of avocado by-products; Table S2. Peak quantification by interpolation and analytical validity of calibration curves used; Table S3. Least squares estimate of regression coefficients for predicting significant variables in response variables studied for UAE and MAE of bioactive compounds in avocado peel by-products; Table S4. Multiple R-squared. Model F-statistic and associated *p*-value and lack-of-fit test statistics from ANOVA for response variables studied for UAE and MAE of bioactive compounds in avocado peel by-products; Figure S1. HPLC-MS full-scan signal obtained in UAE and MAE of avocado by-products.
