# Supplementary material for: Optimization of Extraction Parameters for Phenolics Recovery from Avocado Peels Using Ultrasound and Microwave Technologies
Source: Foods. 2025 Jul 10;14(14):2431. doi: 10.3390/foods14142431 (PMC12295644; doi:10.3390/foods14142431)
Supplement: Supplementary file 1 [file foods-14-02431-s001.zip › foods-3738490-supplementary.pdf]

Table S1. Identification of phenolics and other polar compounds in the UAE and MAE of avocado by-products.

Compound	m/z (-)	Rt (min)	Collision energy (eV)	Dwell	Fragmentor (V)	m/z fragment
Quinic acid	191.1	1.270	40	16	140	108.8
Chlorogenic acid	353.1	4.541	20	16	140	190.9
Procyanidin dimer B1	577.0	5.667	20	16	140	289.1
Epicatechin	289.1	7.414	25	16	140	109.0
Procyanidin trimer C1	865.1	8.660	30	16	140	286.8
Quercetin-O-arabinosyl-glucoside	595.2	9.583	30	16	140	300.1
Quercetin 3-O-glucuronide	477.0	10.554	20	16	140	301.1
Rutin	609.2	10.899	40	16	140	299.9
8-prenylnaringenin	339.2	12.998	40	16	140	183.0
Hydroxyoxooctadecenoic acid	311.1	18.325	40	16	140	183.0

Table S2. Peak quantification by interpolation and analytical validity of calibration curves used.

Compound	Standard	Calibration curve	R^2	LOD (g kg^{-1} dw)	LOQ (g kg^{-1} dw)
Quinic acid	Citric acid	y= 249201.5x-24831.6	0.990	$1.150 \cdot 10^{-2}$	$3.832 \cdot 10^{-2}$
Chlorogenic acid	Chlorogenic acid	y= 243047.4x+237629.5	0.988	0.1986	0.6620
Procyanidin dimer B1	Procyanidin dimer B1	y= 12340785.5x+1075703.7	0.988	0.0783	0.2373
Epicatechin	Epicatechin	y= 58464.8x+377990.5	0.981	0.6910	2.303
Procyanidin trimer C1	Procyanidin dimer B1	y= 12340785.5x+1075703.7	0.988	0.0783	0.2373
Quercetin-3-O-arabinosyl-glucoside	Eriocitrin	y= 143069.5x+894765.4	0.995	0.1849	0.6163
Quercetin 3-O-glucuronide	Eriocitrin	y= 143069.5x+894765.4	0.995	0.1849	0.6163
Rutin	Rutin	y= 54678.5x-31008.7	0.998	$8.238 \cdot 10^{-3}$	$2.746 \cdot 10^{-2}$
8-prenylnaringenin	Naringenin	y= 1447114.4x+626176.6	0.994	$1.945 \cdot 10^{-2}$	$6.488 \cdot 10^{-2}$

Table S3. Least squares estimate of regression coefficients for predicting significant variables in response variables studied for UAE and MAE of bioactive compounds in avocado peel by-products.

		Procyanidin trimer C1			Procyanidin dimer B1			Epicatechin		
		Estimate	SE	*p*	Estimate	SE	*p*	Estimate	SE	*p*
UAE	(Intercept)	4.91	0.22	***	31.09	1.74	***	7.44	0.42	***
	X1	0.08	0.15	ns	1.42	1.15	ns	-0.15	0.28	ns
	X2	0.19	0.15	ns	1.76	1.15	ns	-0.16	0.28	ns
	X3	1.24	0.15	***	11.84	1.15	***	3.08	0.28	***
	X1:X2	-0.26	0.19	ns	-2.07	1.50	ns	0.61	0.37	ns
	X1:X3	0.25	0.19	ns	1.58	1.50	ns	0.10	0.37	ns
	X2:X3	-0.20	0.19	ns	-2.61	1.50	ns	-0.59	0.37	ns
	$X1^2$	-0.08	0.14	ns	-1.04	1.10	ns	-0.04	0.27	ns
	$X2^2$	-0.05	0.14	ns	-0.88	1.10	ns	0.03	0.27	ns
	$X3^2$	-0.06	0.14	ns	-4.02	1.12	ns	-0.61	0.27	*
MAE	(Intercept)	5.40	0.35	***	32.82	2.75	***	7.99	0.31	***
	X1	0.37	0.23	ns	3.62	1.83	ns	0.13	0.20	ns
	X2	-0.02	0.23	ns	0.58	1.81	ns	0.07	0.20	ns
	X3	0.57	0.23	*	5.54	1.82	*	2.04	0.20	***
	X1:X2	-0.05	0.30	ns	1.38	2.38	ns	-0.07	0.27	ns
	X1:X3	-0.67	0.30	*	-4.99	2.38	ns	-0.49	0.27	ns
	X2:X3	-0.28	0.30	ns	-0.85	2.38	ns	-0.25	0.27	ns
	$X1^2$	-0.09	0.23	ns	-0.82	1.80	ns	-0.45	0.20	*
	$X2^2$	-0.10	0.22	ns	-0.91	1.72	ns	-0.32	0.19	ns
	$X3^2$	-0.32	0.22	ns	-2.49	1.77	ns	-0.45	0.20	*

X1: Temperature; X2: Time; X3: % Ethanol. * $p<0.05$; ** $p<0.005$; *** $p<0.001$; ns: no significant.

Table S4. Multiple R-squared. model F-statistic and associated p-value and lack-of-fit test statistics from the ANOVA for response variables studied for UAE and MAE of bioactive compounds in avocado peel by-products.

	Response	R^2	F-statistic	p-value	Lack-of-fit test				
					DF	Sum Sq	Mean Sq	F-statistic	p-value
UAE	TPC	0.609	1.34	0.328	5	30.2	4.2	0.31	0.89
	ABTS	0.661	2.17	0.122	5	62.7	12.54	20.89	0.0023
	Procyanidin trimer C1	0.888	8.84	0.00105	5	1.94	0.39	1.98	0.24
	Procyanidin dimer B1	0.928	14.3	0.000132	5	129	26	2.51	0.168
	Epicatechin	0.929	14.6	0.000121	5	8.9	1.8	4.87	0.054
	Quinic acid	0.591	1.61	0.235	5	6.93e-6	1.39e-6	1.19	0.427
	Chlorogenic acid	0.481	1.03	0.477	5	1.005	0.201	1.27	0.40
	Quercetin 3-O-glucuronide	0.543	1.32	0.333	5	0.2184	0.0437	2.46	0.17
	Rutin	0.534	1.27	0.354	5	2.504	0.501	4.11	0.073
	8-prenylnaringenin	0.264	0.399	0.909	5	0.0720	0.01439	5.52	0.042
MAE	TPC	0.679	2.35	0.0998	5	81.5	22.3	0.90	0.544
	ABTS	0.821	5.09	0.00899	5	0.215	0.0431	23.80	0.0017
	Procyanidin trimer C1	0.624	1.85	0.176	5	6.86	1.372	17.87	0.0033
	Procyanidin dimer B1	0.670	2.26	0.11	5	43.8	8.76	27.03	0.0013
	Epicatechin	0.92	12.9	0.000215	5	5.1	1.01	8.45	0.018
	Quinic acid	0.583	1.55	0.251	5	3.0e-6	5.99e-7	2.72	0.15
	Chlorogenic acid	0.599	1.66	0.22	5	0.616	0.1233	6.46	0.031
	Quercetin 3-O-glucuronide	0.657	2.12	0.128	5	0.1184	0.0237	12.45	0.0075
	Rutin	0.504	1.13	0.423	5	1.404	0.2807	9.64	0.013
	8-prenylnaringenin	0.216	0.306	0.955	5	0.00331	0.000661	0.77	0.61

DF: degrees of freedom; Sq: squares

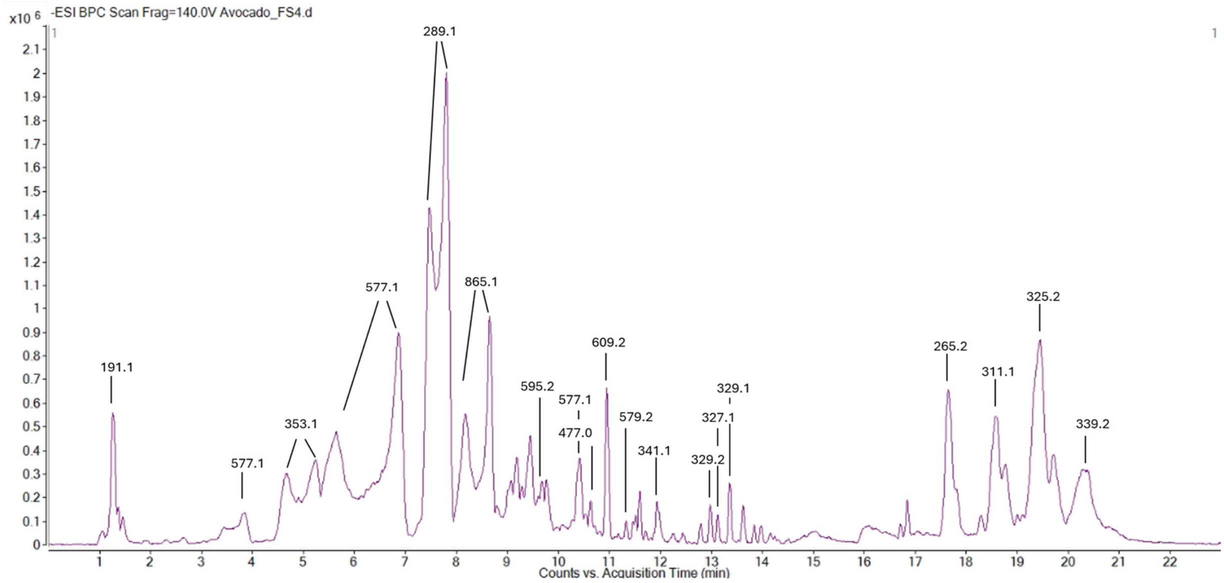

Figure S1. HPLC-MS Full Scan signal obtained in UAE and MAE of avocado by-products.
